# Glucocorticoid therapy reduces ocular hypertension in active moderate-severe thyroid-associated orbitopathy

**DOI:** 10.1186/s12902-022-01153-w

**Published:** 2022-09-23

**Authors:** Chengyang Tang, Liang liang, Xiaoya zheng, Hong Hu, Chun Liu, Jian Long

**Affiliations:** 1 Department of Endocrinology, the First Affiliated Hospital of Chongqing Medical University, Chongqing, 400016 China; 2 Chongqing Key Laboratory of Ophthalmology, and Chongqing Eye Institute, the First Affiliated Hospital of Chongqing Medical University, Chongqing, 400016 China

**Keywords:** Thyroid-associated orbitopathy, Intraocular pressure, Ocular hypertension, Glucocorticoid therapy

## Abstract

**Purpose:**

Ocular hypertension (OHT) is an important clinical feature of thyroid-associated orbitopathy (TAO).While the prevalence and outcome of OHT in TAO remains unclear. This study investigates this in moderate-severe active TAO.

**Methods:**

Sixty-eight patients with active moderate-severe TAO were recruited, 49 of whom were treated with 12-week GC therapy.The clinical and biochemical parameters were collected.Treatment outcomes were evaluated after GC therapy.

**Results:**

The prevalence of OHT was 44.85% in moderate-severe active TAO patients,including 81.97% of mild hypertension, 13.11% of moderate hypertension and 4.92% of severe hypertension.

Clinical and biochemical parameters had no significant difference between OHT patients and non-OHT patients,such as age, sex distributions, smoking status, the kind and the duration of thyroid disease,the duration of eye symptoms and the level of FT3,FT4,TSH, TR-Ab, and Tpo-Ab, Tg-Ab(all *P* > 0.05).

After GC therapy,the intraocular pressure(IOP) in OHT eyes decreased significantly (*P* < 0.05), while IOP in non-OHT eyes remained unchanged (*P* > 0.05).There was no significant difference in CAS and the effective rate of GC therapy between OHT eyes and non-OHT eyes (*P* > 0.05).

**Conclusion:**

In moderate-severe active TAO, the prevalence of OHT was 44.85%, most of which were mild hypertension.OHT was relieved by GC therapy,which had no effect on the efficacy of GC therapy.Our results will enhance physicians' confidence in GC therapy.

## Introduction

Thyroid-associated ophthalmopathy (TAO) is an organ-specific autoimmune inflammatory disease,which is characterized by the inflammatory cellular infiltration, the proliferation of extraocular muscles and fat, and mucopolysaccharide deposition [1, 2].The main manifestations of TAO include eyelid retraction, lid lag, proptosis, extraocular muscle restriction,exposure keratopathy, and optic neuropathy [3, 4].

Recently, increased intraocular pressure(IOP), so called ocular hypertension (OHT), has been found to be an important clinical feature of TAO [5–7].OHT mainly refers to dysfunction of the production and elimination of aqueous humour,as in primary glaucoma [8].While in TAO,the increased episcleral venous pressure due to the increased volume of orbital contents is thought to be the main reason for OHT [9, 10].Whatever,long-term OHT can lead to compression of the retinal fiber layer, eventually to functional vision loss and blindness [11, 12].To date,there are few studies on OHT in TAO,especially regarding its prevalence, risk factors and treatment outcome.Therefore, it is very interesting and worthwhile to study OHT in TAO.

Intravenous glucocorticoids (GC) are considered as the first-line treatment in moderate-severe active TAO patients [13, 14].GC therapy is safe and effective in reducing the clinical activity score (CAS) of TAO and has beneficial effects on diplopia and proptosis due to its anti-inflammatory and anti-immune effects [15, 16].However,the therapeutic effect of GC on OHT has not been reported.On the contrary,GC has been shown to cause OHT in some conditions [17].Therefore concerns about OHT tend to undermine physicians’ confidence in GC therapy.In this regard,we conducted this study to investigate the prevalence and risk factors of OHT and the effect of GC therapy on OHT in moderate-severe active TAO.

## Methods

### Subjects

TAO patients who visited the First Affiliated Hospital of Chongqing Medical University between June 2018 and July 2021 were recruited. The inclusion criteria were as follows:①moderate-severe active TAO patients; and ②age of > 18 years and < 75 years.The exclusion criteria were as follows:①previous history of glucocorticoid therapy or other immunosuppressant, radiotherapy, or surgical decompression surgery; and②previous history of any other disease known to be associated with OHT, such as glaucoma, diabetic or hypertensive retinopathy;③using any drug known to influence IOP.

All subjects agreed to take part in the present study and signed the consent form. The study was approved by the First Affiliated Hospital of Chongqing Medical University Ethical Committee.

### Ophthalmological evaluation

The diagnosis of TAO was following the consensus statement of the European Group on Graves’ Orbitopathy criteria [18]. The activity of TAO was evaluated by the CAS system, which included seven reference items: chemosis, redness of conjunctiva and eyelid, swelling of the eyelid, swelling of the caruncle, spontaneous ache behind the eyeball, ache on attempted upgaze. One symptom added one point, and the CAS score ≥ 3 was classified as an active stage.

All patients were evaluated and followed by an ophthalmologist.External and funduscopic.

exam,visual acuity,IOP (noncontact tonometer CT-1, Topcon, Japan) and exophthalmometry (Ophthalmotosmeter,German) was completed in each follow-up.OCT(TR-KT-0844,Heidelberg engineering,German), VEP (GT-2008 V-1,China), and visual field (Humphrey Field Analyzer 750i,USA) were performed at the time of diagnosis and were repeated when necessary. IOP was measured in primary position for 3 consecutive times and the average value was taken. OHT was defined as an IOP more than 20 mm Hg, with mild hypertension as an IOP of 20–30 mm Hg, moderate hypertension as an IOP of 31–40 mm Hg, severe hypertension as an IOP more than 41 mm Hg.

### Clinical and biochemical measurements

FT3, FT4, and TSH were determined with electro-chemiluminescence method (Unicel DxI 800 Immunoassay System, Beckman Coulter, USA,normal range:FT3 2.01–4.82 pg/ml;FT4 0.59–1.25 ng/dl;TSH 0.56–5.91uIU/ml). TPO-Ab(DXI800, Beckman, USA,normal range:0-9 IU/ml) Tg-Ab (DXI800, Beckman, USA,normal range:0-4 IU/ml) and TR-Ab (Cobas601, Roche, Germany,normal range:0.8–1.8 IU/L) were examined using chemiluminescence immunoassay.

### GC therapy and evaluation of treatment outcome

An intermediate dose of iv glucocorticoids was used, i.e., a starting dose of 0.5 g iv methylprednisolone once weekly for six weeks, followed by 0.25 g once weekly for 6 weeks. Treatment outcome was evaluated immediately after GC therapy by composite index(CI). CI is composed of entirely objective measures: ≥ 2-mm reduction of lid aperture, ≥ 2 point reduction in 7-item CAS, ≥ 2 mm reduction in exophthalmos, ≥ 8°increase of eye muscle duction,improvement of diplopia. Improvement in ≥ 2 features in one eye without deterioration in the other eye might be considered a positive response to treatment.

### Statistical analysis

Statistical analysis was performed using SPSS 22.0 package software. Variables were presented as means ± SD. Means of continuous variables were compared using the Unpaired t-test, or Mann–Whitney Test (when the data were not normally distributed). The percentage differences between groups were compared using χ2 test or Fisher's Exact Test. The differences between groups were considered statistically significant at *P* < 0.05.

## Results

### Clinical and biochemical characteristics of patients

A total of 68 moderate-severe active TAO patients with mean age of 49.97 ± 13.83 years were enrolled in this study, including 28 females and 40 males.The basic characteristics of all patients were shown in Table 1. 64 patients were Grave’s disease treated by anti-thyroid drug or RAI.4 patients were Hashimoto disease treated by L-T4.The mean duration of eye symptoms was 6.89 ± 8.52 months. The mean exophthalmometry was 19.8 ± 3.36 mm in the right eye and 19.53 ± 3.69 mm in the left eye. The mean CAS was 3.85 ± 0.9 in the right eye and 3.85 ± 0.93 in the left eye. 61(44.85%) eyes were OHT, 50(81.97%) eyes were mild hypertension, 8(13.11%) eyes were moderate hypertension,and 3(4.92%) eyes were severe hypertension.

**Table 1:** Basic characteristics of patients

Characteristics	
Age, years	49.97 ± 13.83
Male/Female, n	40/28
GD/HT,n	64/4
Previous thyroid treatment, n	
Anti-thyroid drug	59
RAI	5
Thyroidectomy	0
L-T4	4
Duration of eye symptoms, month	6.89 ± 8.52
Exophthalmometry, R,mm	19.8 ± 3.36
Exophthalmometry, L,mm	19.53 ± 3.69
CAS,R	3.85 ± 0.9
CAS,L	3.85 ± 0.93
IOP	
Non-OHT, n	75
mild hypertension (20-30 mmHg), n	50
moderate hypertension (30-40 mmHg), n	8
severe hypertension (> 40 mmHg), n	3

Data represented as mean ± SD

*HT* Hashimoto disease, *GD* Grave’s disease, *RAI* radioiodine therapy, *L-T4* levothyroxine, *CAS* Clinical activity score, *IOP* Intraocular Pressure, *OHT* Ocular hypertension, *n* number

### The clinical and biochemical characteristics in OHT patients and non- OHT patients

To investigate the effect of clinical and biochemical characteristics on IOP, 68 patients were divided into non-OHT patients (*n* = 30) and OHT patients (*n* = 38) by IOP. As shown in Table 2, there were no significant differences in age, sex distributions, smoking status, the kind and the duration of thyroid disease, and the duration of eye symptoms between the two groups (all *p* > 0.05). Also, there were no significant differences in the level of FT3, FT4, TSH, and thyroid auto-antibodies (TR-Ab, and Tpo-Ab, Tg-Ab) between the two groups(all *p* > 0.05).

**Table 2:** The clinical and biochemical characteristics in OHT patients and non-OHT patients

	Non-OHT patients (*n* = 30)	OHT patients (*n* = 38)	*P*
Age, years	49.76 ± 14.57	50.13 ± 13.44	0.94^*^
Male/Female,n	14/16	25/13	0.14^#^
Current smokers,n	19	21	0.63^#^
The kind of thyroid disease (GD/HT,n)	28/2	36/2	1.0^#^
Duration of thyroid disease,month	13.73 ± 24.11	24.89 ± 48.97	0.26^*^
Duration of eye symptoms,month	6.64 ± 8.88	7.08 ± 8.34	0.63^*^
FT3(pg/ml)	3.61 ± 0.77	3.62 ± 0.5	0.98^*^
FT4(ng/dl)	0.88 ± 0.23	0.93 ± 0.13	0.07^*^
TSH(uIU/ml)	1.19 ± 1.73	1.04 ± 1.83	0.99^*^
TRAb(IU/l)	16.61 ± 12.34	11.88 ± 8.1	0.10^*^
TgAb(IU/l)	127.68 ± 340.04	80.56 ± 221.78	0.40^*^
TpoAb(IU/l)	201.76 ± 339.58	115.82 ± 220.02	0.56^*^

Data represented as mean ± SD

*HT* Hashimoto disease, *GD* Grave’s disease, *OHT* Ocular hypertension, *n* number

^#^P:χ^2^ test; ^*^P:Mann–Whitney U test

*P* < 0.05 were considered statistically significant

### IOP in each eye before and after 12-weeks GC therapy

Ninety-eight eyes of 49 patients who completed GC therapy were divided into non-OHT eyes and OHT eyes by IOP. The Table 3 showed that IOP decreased significantly in OHT eyes (*P* < 0.05),while remained unchanged in non-OHT eyes(*p* > 0.05).

**Table 3:** IOP before and after GC therapy

		Before GC therapy(*n* = 44)	After GC therapy(n = 54)	*P*
IOP	Non-OHT eyes (*n* = 44)	16.66 ± 2.06	16.61 ± 2.3	0.85
	OHT eyes (*n* = 54)	25.34 ± 6.43	21.34 ± 3.98	0.002

Data represented as mean ± SD; P: Mann–Whitney U test

*OHT* Ocular hypertension, *IOP* Intraocular Pressure, *n* number of eyes

*P* < 0.05 were considered statistically significant

### CAS in each eye before and after 12-weeks GC therapy

CAS was evaluated in 49 patients who completed GC therapy before and after GC therapy.As shown in Table 4,there was no significant difference in CAS between non-OHT eyes and OHT eyes before and after GC therapy (*p* > 0.05).

**Table 4:** CAS before and after GC therapy

		Before GC therapy(n = 44)	After GC therapy(n = 54)	P*
CAS	Non-OHT eyes(*n* = 44)	3.86 ± 0.98	1.77 ± 0.91	0
	OHT eyes(*n* = 54)	4.07 ± 0.95	1.98 ± 0.83	0
	P	0.65	0.2	

Data represented as mean ± SD

*CAS* Clinical activity score, *GC* Glucocorticoid, *OHT* Ocular hypertension

P*:Before GC therapy vs after GC therapy

*P*:Non-OHT eyes vs OHT eyes

*P* and P*:Mann–Whitney U test

*P* < 0.05 were considered statistically significant

### The response to GC therapy in OHT eyes and non-OHT eyes

The treatment outcome were evaluated in 49 patients who completed GC therapy. As shown in Table 5, the efficacy of GC therapy had no significant difference between the two groups after GC therapy(*p* > 0.05).

**Table 5:** The response to GC therapy in OHT eyes and non-OHT eyes

	Number of patients response to GC therapy
Non-OHT eyes(*n* = 44)	40
OHT eyes (*n* = 54)	48
p	1.00

*OHT* Ocular hypertension, *GC* glucocorticoid

*P*: Fisher's Exact Test

*P* < 0.05 were considered statistically significant

## Discussion

Our study showed that OHT was a common clinical manifestation of TAO, which was relieved by GC therapy and did not affect the efficacy of GC therapy.This is the first study to report the characteristics of OHT in TAO and the effect of GC therapy on OHT.Our results suggest that OHT is not a contraindication to GC therapy,and GC therapy is also effective in OHT patients.Our results can enhance physician confidence in GC therapy.

Previous studies have shown that the prevalence of OHT in TAO ranges from 3.9% to 25% [19–21].However,in our study,the overall prevalence of OHT was as high as 44.85%. Nina has reported that the IOP in TAO patients depends on the activity and severity of the disease [22]. Therefore, the prevalence of OHT should be higher in patients with moderate-to-severe active TAO than in mild and inactive TAO patients.In our study,CAS was slightly higher in OHT eyes than in non-OHT eyes,but the difference was not statistically significant.We believe that the small number of included patients may account for the lack of difference.Although the prevalence was a little higher in moderate-to-severe active TAO patients in our study,most of them were mild hypertension.Severe hypertension accounted for only 4.92%.

To look for risk factors for OHT, we analyzed clinical or biochemical characteristics. Disappointingly, none of these factors had an effect on IOP in moderate-to-severe active TAO patients,such as age, sex, smoking status, the kind of thyroid disease, the duration of thyroid disease, duration of eye symptoms, the level of FT3, FT4, TSH, and thyroid auto-antibodies.This result was consistent with C allen’s study [7].

GC therapy, as the first-line therapy for TAO,has been showed to improve soft-tissue changes (swelling and redness of eyelids and conjunctiva) and eye-muscle motility (diplopia).While, the effect GC on OHT is not clear in TAO.Of note,our results showed that IOP decreased obviously in OHT eyes after GC therapy.OHT in TAO is thought to be related with the increased episcleral venous pressure due to the increased volume of orbital contents.As is known to all,GC therapy is known to inhibit inflammatory cell infiltration and mucopolysaccharide deposition, thereby reducing orbital content and improving restrictive ocular motility [23, 24].Therefore GC therapy can reduce OHT by its anti-inflammation and anti-immune functions in TAO.Also,our study showed no significant difference in CAS and the efficacy of GC therapy between OHT and non-OHT eyes after GC therapy,which suggests that OHT does not affect the therapeutic effect of GC.

Furthermore,there was no steroid-induced OHT in our study,although this may be a side effect of GC therapy due to the increased aqueous humor outflow resistance [25].We believe that this may be related to the mode of administration of GC, as local administration is more likely to cause OHT [26].

As a preliminary study, our study has several limitations.Firstly,our study only included moderate-to-severe active TAO patients,which may limit the interpretation of the results to some extent. Secondly, restrictive ocular motility in TAO may lead to a transient elevation of IOP in upgaze and sometimes in primary position [27].Therefore,in our study,measuring IOP only in primary position may increase the prevalence of OHT.However, we believe that this does not affect the comparison of results before and after GC treatment because the IOP is measured in the same way. Finally, the sample size was relatively small due to the strict selection standards. Therefore further studies may be need to complement and extend these preliminary results.

## Conclusion

In moderate-severe active TAO, the prevalence of OHT was 44.85%, most of which were mild hypertension.OHT was relieved by GC therapy,which had no effect on the efficacy of GC therapy.Our results will enhance physicians' confidence in GC therapy.

## Data Availability

The datasets used and/or analysed during the current study available from the corresponding author on reasonable request.
